# Heterosis in Early Maize Ear Inflorescence Development: A Genome-Wide Transcription Analysis for Two Maize Inbred Lines and Their Hybrid

**DOI:** 10.3390/ijms150813892

**Published:** 2014-08-11

**Authors:** Haiping Ding, Cheng Qin, Xirong Luo, Lujiang Li, Zhe Chen, Hongjun Liu, Jian Gao, Haijian Lin, Yaou Shen, Maojun Zhao, Thomas Lübberstedt, Zhiming Zhang, Guangtang Pan

**Affiliations:** 1 Maize Research Institute of Sichuan Agricultural University/Key Laboratory of Biology and Genetic Improvement of Maize in Southwest Region, Ministry of Agriculture, Chengdu 611130, China; E-Mails: dinghp@sicau.edu.cn (H.D.); cheng.qin.sicau@gmail.com; (C.Q.); lilujiang2014@gmail.com (L.L.); chenzhe1024@gmail.com (Z.C.); lhj20305@gmail.com (H.L.); gaojian8888@gmail.com (J.G.); linhj521@gmail.com (H.L.); shenyaou@gmail.com (Y.S.); 2 Zunyi Academy of Agricultural Sciences, Zunyi 563102, China; E-Mail: luoxirong2014@gmail.com; 3 Life Science College, Sichuan Agricultural University, Ya’an 625014, China; E-Mail: toxic.xc@gmail.com; 4 Department of Agronomy, Iowa State University, Ames, IA 50011, USA; E-Mail: thomasl@iastate.edu

**Keywords:** maize (*Zea mays* L.), heterosis, DGE (digital gene expression), differentially expressed genes, multiple molecular mechanisms

## Abstract

Heterosis, or hybrid vigor, contributes to superior agronomic performance of hybrids compared to their inbred parents. Despite its importance, little is known about the genetic and molecular basis of heterosis. Early maize ear inflorescences formation affects grain yield, and are thus an excellent model for molecular mechanisms involved in heterosis. To determine the parental contributions and their regulation during maize ear-development-genesis, we analyzed genome-wide digital gene expression profiles in two maize elite inbred lines (B73 and Mo17) and their F_1_ hybrid using deep sequencing technology. Our analysis revealed 17,128 genes expressed in these three genotypes and 22,789 genes expressed collectively in the present study. Approximately 38% of the genes were differentially expressed in early maize ear inflorescences from heterotic cross, including many transcription factor genes and some presence/absence variations (PAVs) genes, and exhibited multiple modes of gene action. These different genes showing differential expression patterns were mainly enriched in five cellular component categories (organelle, cell, cell part, organelle part and macromolecular complex), five molecular function categories (structural molecule activity, binding, transporter activity, nucleic acid binding transcription factor activity and catalytic activity), and eight biological process categories (cellular process, metabolic process, biological regulation, regulation of biological process, establishment of localization, cellular component organization or biogenesis, response to stimulus and localization). Additionally, a significant number of genes were expressed in only one inbred line or absent in both inbred lines. Comparison of the differences of modes of gene action between previous studies and the present study revealed only a small number of different genes had the same modes of gene action in both maize seedlings and ear inflorescences. This might be an indication that in different tissues or developmental stages, different global expression patterns prevail, which might nevertheless be related to heterosis. Our results support the hypotheses that multiple molecular mechanisms (dominance and overdominance modes) contribute to heterosis.

## 1. Introduction

Heterosis, or hybrid vigor, refers to the phenomenon in which progeny of two inbred lines (hybrids) exhibit enhanced agronomic performance such as biomass production, growth rate, fertility, and disease resistance relative to both parents [1]. Heterosis has been extensively used in agriculture, especially in the breeding of maize and rice. For example, it is estimated that approximately 95% of the United States maize acreage and 55% of rice acreage in China are planted with hybrids. Furthermore, hybrid maize technology for large-scale production has a yield advantage of 15% over the elite inbred varieties. Though the concept of heterosis has been introduced over 100 years ago and different genetic models considered [1,2,3], the genetic and molecular basis of heterosis remains elusive.

Three classical genetic hypotheses to explain heterosis have been proposed: the dominance, overdominance, and epistasis hypotheses. The dominance hypothesis states that deleterious recessive alleles cause inbreeding depression. A cross of two inbred parents will benefit from complementation of these deleterious alleles and will display a superior phenotype [2,3,4]. The over-dominance hypothesis refers to allelic interactions at one or multiple heterozygous genes resulting in superior trait expression compared to the better parent [1,5]. Both hypotheses may be insufficient to explain the molecular mechanism for heterosis [6]. Epistasis hypothesis refers to interactions of alleles at different loci from two parents in F_1_ hybrids, leading to heterosis [7,8].

Recently, genetic and molecular studies provided experimental support for above-mentioned hypotheses. Using molecular markers in segregating populations, quantitative trait locus (QTL) mapping studies provided support for the dominance [9], over-dominance [10], and epistasis [7,8] models. With the advent of genomic methods to assay genome-wide patterns of gene expression, recent studies indifferent tissues and developmental stages of model and crop plants (such as maize, rice, wheat, *Populus*, and *Arabidopsis*) have determined the roles of different gene expression [11,12,13,14,15,16,17,18,19,20,21,22,23,24,25,26,27,28,29,30,31,32], small RNAs [33,34,35,36], and epigenetic regulation [37,38], including circadian-mediated metabolic pathways [39], in heterosis. For instance, additive gene expression was prevalent in some studies of maize [14,26,32], and there were varying numbers of genes that exhibited a non-additive behavior in other studies [12,17,18,19,20,21,22,23,24,25,26,27,29,30,35].

Maize immature ear inflorescences show heterosis in ear architectural traits [40], and significant positive correlations between grain yield and ear architectural traits, such as ear length, kernel row number, number of kernels per row, kernel number density, and cob diameter have been reported [40], which suggests that each of these components contributes to greater yields. A thorough knowledge of the genes affecting the various components and their interactions will facilitate our understanding of the molecular basis of heterosis of grain yield. In this study, we applied a highly effective approach of high throughout deep sequencing to identify genes, which are highly expressed in maize elite inbred lines of B73 and Mo17, and their F_1_ hybrid (B73 × Mo17) at an early stage of ear inflorescences development. We provide first molecular evidence that regulatory mechanisms underlying the phenomenon of heterosis are very early active in maize ear development. Furthermore, we also found that differentially expressed genes between hybrids and their parents can be involved in certain regulatory networks, which suggested that complicated gene networks might be underlying heterosis. Results of the present study might help promote further understanding of mechanisms underlying heterosis.

## 2. Results

### 2.1. Statistics and Analysis of Library Sequencing

Here, we sequenced three ear digital gene expression (DGE) libraries from two inbred parents (B73 and Mo17) and their F_1_ hybrid (B73 × Mo17) using massively parallel sequencing on the Illumina platform at BGI-Shenzhen, China (Table 1 and Table S1**)**. A total of approximately 4.2 million raw tags per library with 259,890 distinct tag sequences were identified. After data-processing steps (see Materials and Methods), the total number of filtered, high-quality clean tags was almost the same in three libraries. Furthermore, the F_1_ library had the highest number of distinct tags (259,282), followed by the B73 (242,184), and Mo17 (239,963) libraries (Table 1 and Table S1). These distinct tags and their genomic frequency as well as the raw data were deposited in NCBI Sequence Read Archive (SRA) database with the accession number (PRJNA248701). Copy numbers of most of the distinct tags (over 77%) ranged from 1–5. However, a small number of distinct tags (less than 3.29%) with a frequency higher than 100 make up over 62% of all clean tags in all three libraries (Figure S1).

When sequencing depths reach 1 million total tags, the number of novel distinct tags discovered dropped dramatically in all three libraries (Figure S2A). From that point, increasing sequence depth results in a slow and stable accumulation of new distinct tags indicating that sequencing has reached saturation. Moreover, as shown in Figure 2B, when the total tag number in B73 reached 1 million, the increase of identified genes started to level out, and stabilized when the number of tags reached 3 million. Next, the level of gene expression was determined by calculating the number of unambiguous clean tags for each gene and then normalized to the number of transcripts per million tags (TPM). The Mo17 and F_1_ data showed a similar trend (Figure S2). This suggests that only few more distinct genes would be identified when the total clean tag number reached a certain value.

**Table 1:** Summary statistics from mapping digital gene expression (DGE) sequencetags to the maize B73 reference genome.

Class	Summary	F_1_	B73	Mo17
Raw Data	Total	4,200,000	4,200,000	4,200,000
	Distinct Tags	272,402	254,882	252,386
Clean Tags	Total number	4,176,622	4,176,825	4,176,752
	Distinct Tags number	259,282	242,184	239,963
All Tags Mapping to Genome	Total number	2,766,685	2,979,615	3,127,520
	Total % of clean tags	66.24%	71.34%	74.88%
	Distinct Tags number	134,449	134,378	141,476
	Distinct Tags % of clean tags	51.85%	55.49%	58.96%
Unambiguous Tags Mapping to Genome	Total number	2,452,293	2,637,419	2,804,389
	Total % of clean tags	58.71%	63.14%	67.14%
	Distinct Tags number	119,316	118,719	125,467
	Distinct Tags % of clean tags	46.02%	49.02%	52.29%
All Tags Mapping to Genes	Number	24,629	24,078	24,198
	% of ref. genes	75.69%	74%	74.36%
Unambiguous Tags Mapping to Genes	Number	21,372	20,784	20,938
	% of ref. genes	65.68%	63.87%	64.35%
Unknown Tags	Total number	894,503	721,786	561,476
	Total % of clean tags	21.42%	17.28%	13.44%
	Distinct Tags number	85,152	70,700	61,016
	Distinct Tags % of clean tags	32.84%	29.19%	25.43%

### 2.2. Mapping Tags to the Maize Reference Genome

We used SOAP2 software [41] to map all distinct tags to the maize reference genome (B73 RefGen_v2) [42]. Mapping results showed that 51.85%, 55.49% and 58.96%, respectively, of distinct clean tags mapped to the reference database (sense or anti-sense), and 46.02%, 49.02%, and 52.29%, respectively, of the distinct clean tags mapped unambiguously to the reference genes (Table 1 and Table S1). Out these, we identified 20,784–21,372 genes expressed in the three genotype comparison (Table S2), our analysis revealed 17,128 genes expressed in all samples and 22,789 genes expressed collectively in the present study (Table S3). In total, 32.84%, 29.19%, and 25.43% of all distinct clean tags for F_1_, B73, and Mo17 data sets, respectively, did not map to the reference maize genome sequence or associated transcripts (Table 1 and Table S1). These non-mapped tags most likely represent regions where the maize reference sequence is incomplete [43] or there are differential mRNA processing events for most maize genes, such as alternative splicing [44]. Only 0.03% of non-mapped tags matched maize chloroplast or mitochondrial genome sequences (Table S1). Because Solexa sequencing can distinguish transcripts originating from both DNA strands, we found evidence for bidirectional transcription in 14,012–14,420 of all detectable overlapping genes and 1031–1119 antisense-stand specific transcripts based on the strand-specific nature of the sequenced tags (Table S2). By comparison, the ratio of sense to antisense strand of the transcripts was approximately 1.3:1 for all libraries. As summarized in Figure S3 and Table S3, most expressed genes (approx. 19,965) are represented in fewer than one hundred copies and only a small proportion of genes are highly expressed (Table S3). We map the clean tags, which cannot be mapped to mRNA, mitochondria and chloroplasts, to the whole genome, providing start positions that could be uniquely mapped by those tags (Table S4).

### 2.3. Different Gene Expression Analysis

**Figure 1:**
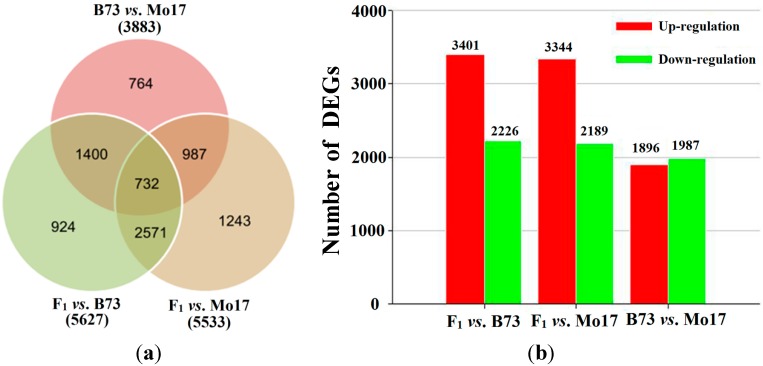
Differentially expressed genes in the maize heterotic cross. Venn diagram (**a**) and statistical analysis (**b**) of differentially expressed genes among inbred parents and their F_1_ hybrid.

A total of 32,973 significantly changed tags entities were detected among three genotypes (see Materials and Methods) (Figure S4A,C,E and Table S5). Then, the processed DGE data were used to determine different gene expression between inbred parents or between parental lines and their F_1_ hybrid and we identified 8621 out of 22,789 genes that were differentially expressed among B73, Mo17, and their hybrid, representing 38% of the ear digital gene expression (Figure 1A). Among them, 3401 (3344) and 2226 (2189) genes were significantly up-regulated and down-regulated, respectively, in the F_1_ compared to B73/Mo17 (Figure 1B, Figure S4B,D and Table S6). The comparison between parental line libraries also revealed significant variation in expression. A total of 3883 genes, including 1896 up-regulated and 1987 down-regulated genes, were identified in B73 compared to Mo17 (Figure 1B, Figure S4F and Table S6).

**Figure 2:**
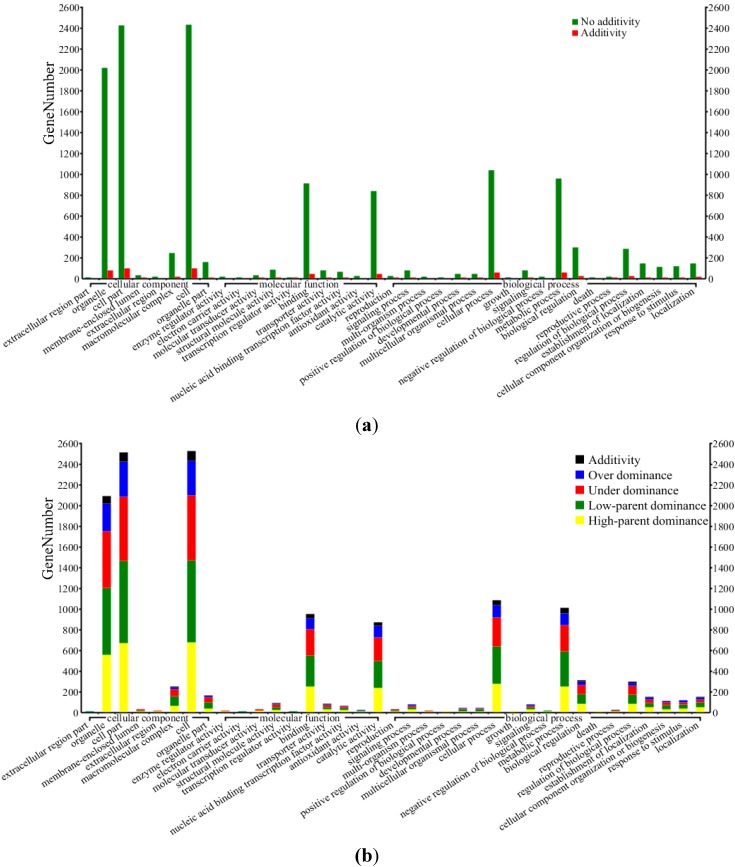
Functional categories of differentially expressed genes. Overall differentially expressed genes (**a**) and non-additive expressed genes (**b**) from B73 × Mo17 cross.

**Figure 3:**
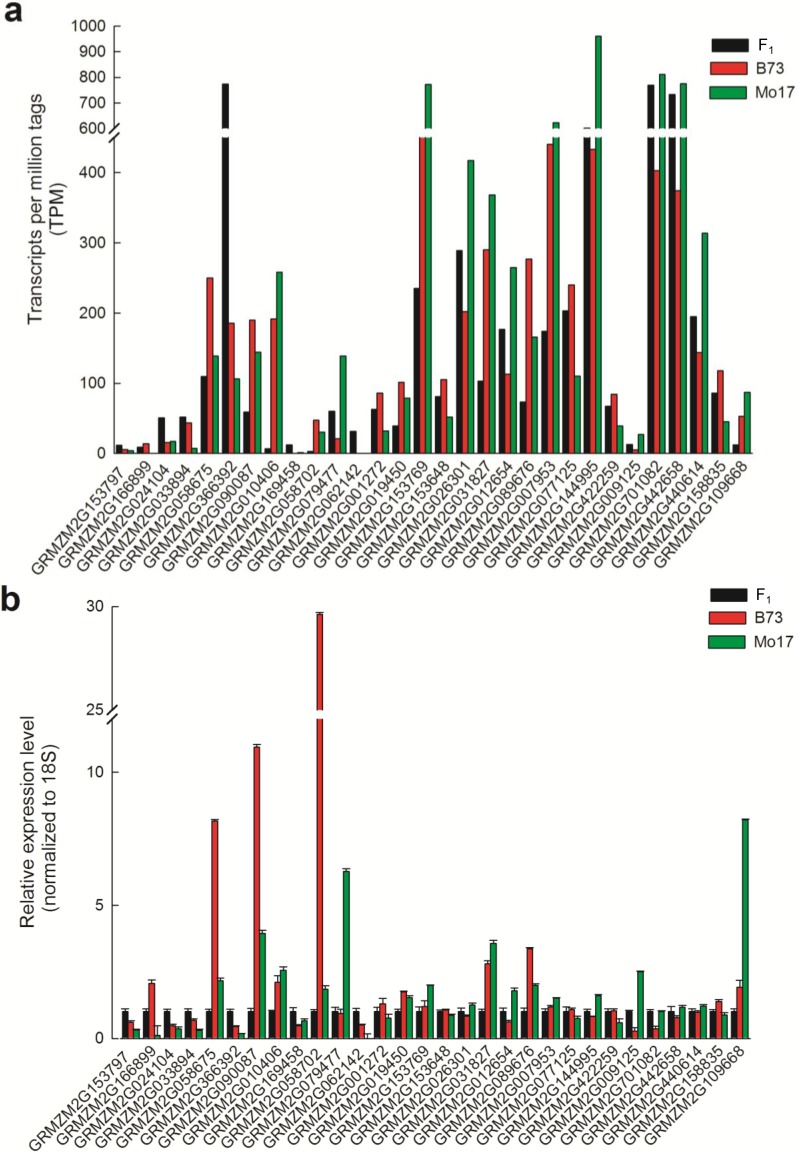
Expression of 12 differentially expressed gene from arginine and proline metabolism, pyruvate metabolism, and purine metabolism in the maize heterotic cross. The expression pattern of 12 genes detected by digital gene expression platform(**a**) and quantitative Real-Time PCR (qRT-PCR) (**b**). The black, red, and green bars in graph b depicted the stem-loop qRT-PCR relative expression level ± standard error of three replicates for each gene inB73, Mo17 and their hybrid.

We further investigated the mode of gene action for these different genes (Table S6). 8.9% (767 of 8621) exhibited an expression pattern that was not distinguishable from additivity, while the other 91.1% (7854 of 8621) genes showed non-additive expression patterns (Figure 2A). The non-additive differentially expressed genes from the cross were further classified into four distinct classes: high-parent dominance (1984), low-parent dominance (2559), over-dominance (1085), and under-dominance (1963) (Figure 2B and Table 2). A sample of 30 differentially expressed genes was randomly selected for validation by qRT-PCR. The trends in the expression of these genes detected by DGE were consistent (29 genes) or partially (1 gene) consistent with those determined in qRT-PCR analyses (Figure 3). These findings are consistent with a recent study in maize [31] or rice [16] that supports the involvement of multiple modes (dominance and overdominance) of gene action in association with heterosis.

**Table 2:** Statistical analysis of differentially expressed genes (FDR ≤ 0.001 and absolute value of log_2_Ratio ≥ 1).

Hybrid Cross	B73 × Mo17
**Total**	8621
**Additivity**	767
**Non Additivity**	7854
**High-Parent Dominance**	1984
**Low-Parent Dominance**	2559
**Over Dominance**	1085
**Under Dominance**	1963
**Other**	263

F_1_ represents the hybrid line; P, paternal line, B73; and M, maternal line, Mo17. Additivity, F_1_≈ 1/2(P + M); non-additivity, F_1_ > 1/2 (P + M) or F_1_ < 1/2 (P + M). High-parent dominance (HPD), F_1_ ≈ P > M or F_1_ ≈ M < P; low-parent dominance (LPD), F_1_≈ P < M or F_1_≈ M < P; over-dominance (ODO), F_1_ > P and F_1_ > M; under-dominance (UDO), F_1_ < P and F_1_ < M.

### 2.4. Functional Enrichment Analysis for Differentially Expressed Genes Using Gene Ontology (GO)

To gain a better understanding of the functional roles of different genes between inbred parents or between parental lines and their F_1_ hybrid, we looked for gene enrichment regarding the Gene Ontology (GO) cellular component, molecular function and biological process categories [44,45]. Functional-annotations from the maize genome (http://maizesequence.org) were used for functional classification of the 8621 different genes and performed by the official web-based tools for searching and browsing the Gene Ontology database (AmiGO) (http://www.geneontology.org/) [45] (Figure 2 and Table 3). We found that different genes showing differential expression patterns were mainly enriched in five cellular component categories (organelle, cell, cell part, organelle part and macromolecular complex), five molecular function categories (structural molecule activity, binding, transporter activity, nucleic acid binding transcription factor activity and catalytic activity), and eight biological process categories (cellular process, metabolic process, biological regulation, regulation of biological process, establishment of localization, cellular component organization or biogenesis, response to stimulus and localization) (Table 3). We further classified different genes in more detail based on Gene Ontology (GO) by AmiGO [45] (Table S7–S9). These different genes in GO annotation analysis of cellular component showed significant enrichment in the following components: non-membrane-bound organelle (6.40 × 10^−4^), intracellular non-membrane-bound organelle (6.40 × 10^−4^), nucleus (2.56 × 10^−3^), ribosome (3.34 × 10^−2^), and macromolecular complex (4.27 × 10^−2^). In the GO biological process enrichment analysis, there are five significant GO terms (FDR corrected *p*-value < 0.05). Eighty percent were macromolecular synthesis-related, such as cellular protein metabolic process, cellular macromolecule metabolic process, protein metabolic process, and macromolecule metabolic process (Table 4).

**Table 3:** Functional classification of different genes between inbred parents or between parental lines and their F_1_ hybrid.* indicated the significant enrichment in functional classification.

Functional Categories	Additivity	Non-Additivity	HPD	LPD	UDO	ODO	Other
**Cellular Component**							
Extracellular Region Part	1	3	0	2	0	1	0
Organelle *	181	2077	544	650	542	271	70
Cell Part	221	2499	655	793	624	339	88
Membrane-Enclosed Lumen	1	24	3	9	10	1	1
Extracellular Region	1	13	1	5	2	5	0
Macromolecular Complex *	16	245	58	89	71	16	11
Cell	221	2510	660	794	628	340	88
Organelle Part	16	155	31	57	47	14	6
**Molecular Function**							
Enzyme Regulator Activity	3	12	3	2	3	4	0
Electron Carrier Activity	0	3	0	1	2	0	0
Molecular Transducer Activity	3	24	7	5	9	2	1
Structural Molecule Activity	3	80	17	27	28	4	4
Transcription Regulator Activity	2	6	0	1	2	2	1
Binding	75	940	241	297	258	108	36
Transporter Activity	9	76	25	19	20	9	3
Nucleic Acid Binding Transcription Factor Activity	2	58	18	19	15	2	4
Antioxidant Activity	0	17	5	3	3	6	0
Catalytic Activity	67	865	229	263	226	111	36
**Biological Process**							
Reproduction	0	21	3	8	5	3	2
Signaling Process	5	70	24	20	16	7	3
Multi-Organism Process	0	8	4	2	1	1	0
Positive Regulation of Biological Process	0	3	2	1	0	0	0
Developmental Process	2	38	2	21	9	4	2
Multicellular Organismal Process	2	39	4	20	9	4	2
Cellular Process	71	1079	269	357	281	125	47
Growth	0	5	1	2	1	1	0
Signaling	5	70	24	20	16	7	3
Negative Regulation of Biological Process	2	12	2	6	3	1	0
Metabolic Process	70	999	242	338	253	118	48
Biological Regulation	18	303	77	94	88	28	16
Death	2	6	2	1	2	1	0
Reproductive Process	0	15	1	5	5	2	2
Regulation of Biological Process	17	289	76	88	84	26	15
Establishment of Localization	15	143	41	44	33	19	6
Cellular Component Organization or Biogenesis	10	106	24	38	29	10	5
Response to Stimulus	4	112	33	37	22	19	1
Localization	16	145	41	45	33	19	7

**Table 4:** Significant GO terms of DGs in the gene ontology (GO) annotation analysis of cellular component and biological process. ^a^ *p*-values calculated using a hypergeometric test-determines if the number of times that a GO term appears in the cluster is significant, relative to its occurrence in the genome.

GO Term	Definition	FDR Corrected *p*-Value ^a^
Cellular Component		
GO:0043228	Non-membrane-bounded organelle	6.40 × 10^−4^
GO:0043232	Intracellular non-membrane-bounded organelle	6.40 × 10^−4^
GO:0005634	Nucleus	2.56 × 10^−3^
GO:0005840	Ribosome	3.34 × 10^−2^
GO:0032991	Macromolecular complex	4.27 × 10^−2^
Biological Process		
GO:0044267	Cellular protein metabolic process	4.22 × 10^−3^
GO:0044260	Cellular macromolecule metabolic process	8.84 × 10^−3^
GO:0019538	Protein metabolic process	3.30 × 10^−2^
GO:0009987	Cellular process	3.37 × 10^−2^
GO:0043170	Macromolecule metabolic process	4.59 × 10^−2^

### 2.5. The Expression Patterns of Biological Macromolecular Synthesis-Related Genes in the B73 × Mo17 Hybrid

To investigate pathways in which different genes were involved and enriched, pathway analysis was performed using the KOBAS 2.0 web tool [46]. 2935 out of 5627 different genes were involved in 122 pathways between F_1_ and B73, 2877 out of 5533 different genes were involved in 121 pathways between F_1_ and Mo17 (Table S10), Among them, the majority were present in ribosome, spliceosome and various metabolic pathways (such as arginine and proline metabolism, purine metabolism, glycolysis/gluconeogenesis, and biosynthesis of alkaloids derived from histidine and purine, *etc.*) (Table S10). *q*-value estimates [47] revealed only four pathways showing significance (*q* < 0.05): ribosome (*q* = 1.58 × 10^−3^), arginine and proline metabolism (*q* = 1.58 × 10^−3^), spliceosome (*q* = 1.63 × 10^−2^) and pyruvate metabolism (*q* = 3.80 × 10^−2^) (Table 5 and Table S11), and the first two pathways showed extreme significance (*q* < 0.01). Of the top 10 differentially expressed genes enriched in pathways between parental lines and their F_1_ hybrid, it should be noted that the purine metabolism pathway was the third largest pathway, following the ribosome pathway, although it does not reach the significance level (Table 5 and Table S11).

**Table 5:** Top 10 pathways differentially expressed genes enriched in between parental lines and their F_1_ hybrid.

Pathway ^a^	Different Genes with Pathway Annotation (4312)				*p* -Value ^b^	*q* -Value ^c^	Pathway ID
	F_1_ *vs.* B73	F_1_ *vs.* Mo17	Overlap	Total (%)			
Ribosome **	148	129	89	188 (4.68%)	2.47 × 10^−5^	1.58 × 10^−3^	ko03010
Arginine and Proline Metabolism **	37	34	25	46 (1.11%)	2.59 × 10^−5^	1.58 × 10^−3^	ko00330
Spliceosome *	145	136	87	194 (4.82%)	4.01 × 10^−4^	1.63 × 10^−2^	ko03040
Pyruvate Metabolism *	32	41	25	49 (1.69%)	6.28 × 10^−4^	3.80 × 10^−2^	ko00620
Proteasome	33	25	19	39 (0.90%)	2.00 × 10^−3^	6.09 × 10^−2^	ko03050
Pentose Phosphate Pathway	16	24	13	27 (0.63%)	1.66 × 10^−3^	6.70 × 10^−2^	ko00030
Purine Metabolism	64	72	39	97 (2.25%)	9.34 × 10^−4^	7.50 × 10^−2^	ko00230
RNA Degradation	43	49	31	61 (1.41%)	4.38 × 10^−3^	8.84 × 10^−2^	ko03018
Porphyrin and Chlorophyll Metabolism	21	16	11	26 (0.60%)	3.82 × 10^−3^	9.32 × 10^−2^	ko00860
Glycolysis/Gluconeogenesis	49	57	31	75 (1.74%)	8.38 × 10^−3^	1.04 × 10^−1^	ko00010

^a^ Pathwayanalysisbased on KOBAS 2.0 (Mao *et al.* 2005; Wu *et al.* 2006; Xie *et al.* 2011) [46,48,49]; ^b^ *p*-value in hypergeometric test; ^c^ The *q*-value is similar to the well known *p*-value, except it is a measure of significance in terms of the false discovery rate rather than the false positive rate (Storey *et al.* 2003) [47]; the top 10 pathways with *q*-value are listed; * pathway with *q*-value < 0.05 is considered as significant; ** pathway with *q*-value < 0.01 is considered as extreme significant.

In the four significant pathways, 188 different genes (4.68%) were detected in the ribosome pathway, 46 different genes (1.11%) in the arginine and proline metabolism pathway, 194 (4.82%) in the spliceosome pathway and 49 (1.69%) in the pyruvate metabolism pathway. Approximately 50% of different genes in every pathway were differentially expressed between inbred and hybrid (Table 5). In the two significant metabolic pathways, almost all (202 out of 204) different genes showed non-additive expression patterns (HPD, UDO, LPD, or UDO). Seventeen of the 46 different genes in the arginine and proline metabolism pathway were up-regulated (with expression pattern of HPD or ODO) in the F_1_ hybrid, and 26 of 46 were down-regulated (with expression pattern of LPD or UDO). As compared with parental lines, the transcriptional level of one different gene in the F_1_ hybrid (GRMZM2G062142) encoding ornithine carbamoyltransferase (OTC) was up-regulated by 7- and 11.6-fold, respectively. The gene (GRMZM2G169458) encoding fatty aldehyde dehydrogenase 1 and another gene (GRMZM2G366392) encoding *S*-adenosylmethionine decarboxylase proenzyme (SAMDC) showed 4.7-/3.4-fold and 2.1-/2.9-fold higher expression in F_1_ hybrid than the parental lines, respectively. Simultaneously, the expression levels of another different gene in the F_1_ hybrid (GRMZM2G035042) encoding IMP dehydrogenase/GMP reductase were down-regulated by 8.5- and 9.3-fold, respectively, comparing with parental lines, another gene (GRMZM2G010406) encoding argininosuccinate synthase showed significant down-regulation expression in F_1_ hybrid. Moreover, 50 different genes involved in the pyruvate metabolism included genes encoding glyoxylatereductase (GRMZM2G166899), phosphoenolpyruvate carboxylase1 (PEPCase 1, GRMZM2G083841), oxidoreductase (GRMZM2G118770), and Pyruvate, orthophosphate dikinase 1 (GRMZM2G097457). (Table S8**)**.

### 2.6. Resolving Transcription Factors (TFs) among Differentially Expressed Genes

A primary objective was to identify genes that encode TFs and to determine their modes of gene action. To test this, we retrieved putative orthologs of maize genes in our differently expressed data based on information from the Ensembl Compara gene trees [50] at maizesequence.org and gramene.org [51]. We then queried known *Arabidopsis* TFs in the database of *Arabidopsis* Transcription Factors (http://datf.cbi.pku.edu.cn/) and identified 435 maize different genes with sequence similarities to *Arabidopsis* TFs between parental lines and their F_1_ hybrid libraries (Table S12). We further interrogated these different genes using gene ontologies, InterPro domains, and known maize annotations. Of the 435 putative TFs, 11 exhibited additive gene action, and the majority of these TFs (*n* = 424) detected in this study exhibited nonadditive modes of gene expression. Most of these genes exhibited low-parent dominance (*n* = 138), high-parent dominance (*n* = 112), and underdominance (*n* = 112). However, overdominance (*n* = 42) and other gene action (*n* = 19) were also observed.

We also surveyed the differential expression of TFs across a wide range of transcript abundance in hybrid here and a mutant in *RAMOSA3* (*RA3*) gene reported in a previous study [52], the latter of which showed an increased branching phenotype resulting from a loss of determinacy of basal spikelet pair meristems. A total of 39 differentially expressed putative TFs in our dataset were identified, and exhibited all nonadditive modes of gene expression (Table 6). Moreover, these TFs were also differentially expressed over a wide range of abundances in *ra3* mutants [52]. These TFs includes several kinds of members of TF families associated with functions in development and meristem maintenance or identity (NAC, YABBY, GRAS and TCP), while others have roles in hormone-mediated or stress-mediated signaling by auxin (AUX/IAA), brassinosteroids (BES), or ethylene and stress (AP2/ERF). Among the 42 differentially expressed TFs, eight were characterized as AP2/ERF family proteins (Table 6). Therefore, these TFs possibly not only contribute to heterosis, but also provide insight into genetic control of branching.

**Table 6:** Differentially expressed maize genes were identified as putative TFs.

Maize Gene ID	Annotation ^a^	TF Family	Significant Pattern ^b^	Expression Model ^c^	Regulated in *ra3* Mutants ^d^
GRMZM2G106673	B3-domain TF	B3	Mo17 < F_1_ ≈ B73	HPD	Up
GRMZM2G177046	Ocs element-binding factor 1	bZIP	Mo17 < F_1_ ≈ B73	HPD	Up
GRMZM2G102514	BES1/BZR1 protein	BES	Mo17 < F_1_ ≈ B73	HPD	Down
GRMZM2G172657	Uncharacterized	GRAS	Mo17 < F_1_ ≈ B73	HPD	Down
GRMZM2G115357	IAA24	AUX/IAA	Mo17 < F_1_ ≈ B73	HPD	Up
GRMZM2G181376	Uncharacterized	–	B73 < F_1_ ≈ Mo17	HPD	Down
GRMZM2G173534	Inducer of CBF expression 2	–	B73 < F_1_ ≈ Mo17	HPD	Up
GRMZM2G173124	Zinc finger	Znf-C3H1	B73 ≈ F_1_ < Mo17	LPD	Up
GRMZM2G138886	Cyclin B2	Cyclin	B73 ≈ F_1_ < Mo17	LPD	Up
GRMZM2G020054	Uncharacterized	AP2/ERF	Mo17 ≈ F_1_ < B73	LPD	Up
GRMZM2G055243	KNOX class 2 protein	KNOX	Mo17 ≈ F_1_ < B73	LPD	Up
GRMZM2G089995	Ethylene responsive	AP2/ERF	Mo17 ≈ F_1_ < B73	LPD	Up
GRMZM2G079825	Pathogenesis-related	AP2/ERF	Mo17 ≈ F_1_ < B73	LPD	NS
GRMZM2G310368	Ethylene responsive	AP2/ERF	Mo17 ≈ F_1_ < B73	LPD	Up
GRMZM2G132185	Pathogenesis-related	AP2/ERF	Mo17 ≈ F_1_ < B73	LPD	NS
GRMZM2G149940	B3 DNA binding domain	B3	Mo17 ≈ F_1_ < B73	LPD	Up
GRMZM2G003927	Ramosa1	Znf-C2H2	B73 ≈ F_1_ < Mo17	LPD	Up
GRMZM2G447406	Progesterone receptor	–	B73 ≈ F_1_ < Mo17	LPD	Down
GRMZM2G102218	YABBY protein	YABBY	Mo17 ≈ F_1_ < B73	LPD	Up
GRMZM2G136769	Ubiquitin-associated	Ubiquitin	Mo17 ≈ F_1_ < B73	LPD	Up
GRMZM2G165972	Heat shock factor (HSF)-type	HSF	Mo17 ≈ F_1_ < B73	LPD	Down
GRMZM2G140474	Tyrosine protein kinase	–	Mo17 ≈ F_1_ < B73	LPD	Up
GRMZM2G422205	Zinc finger	Znf-C3H1	Mo17 ≈ F_1_ < B73	LPD	
GRMZM2G171468	Uncharacterized	MYB	Mo17 ≈ F_1_ < B73	LPD	Down
AC206951.3_FG017	Ethylene-responsive element binding protein 2	ERF	Mo17 < B73 < F_1_	ODO	Up
GRMZM2G081012	Transcription initiation factor IID, 18 kD subunit family protein	TFIID-18	B73 ≈ Mo17 < F_1_	ODO	Up
GRMZM2G014653	NAC protein 48	NAC	B73 ≈ Mo17 < F_1_	ODO	Up
GRMZM2G127379	NAM containing	NAC	B73 ≈ Mo17 < F_1_	ODO	Up
GRMZM2G061487	DRE binding factor 1	AP2/ERF	F_1_ < Mo17 < B73	UDO	Up
GRMZM2G431157	Zinc finger, C_2_H_2_-type	Znf_C2H2-like	F_1_ < B73 ≈ Mo17	UDO	Up
GRMZM2G307119	Branched silkless1	AP2/ERF	F_1_ < B73 ≈ Mo17	UDO	Up
GRMZM2G144275	bHLH transcription factor	HLH	F_1_ < B73 ≈ Mo17	UDO	Up
GRMZM2G132367	HDZipI-1	HD-Zip	F_1_ < B73 ≈ Mo17	UDO	Up
GRMZM2G105266	Pathogenesis-related	AP2/ERF	F_1_ < B73 ≈ Mo17	UDO	NS
GRMZM2G381395	DNA-directed RNA polymerase	–	F_1_ < B73 ≈ Mo17	UDO	Down
GRMZM2G453424	Uncharacterized	HRDC_like	F_1_ < B73 ≈ Mo17	UDO	Up
GRMZM2G118113	DNA-directed RNA polymerase II 8.2 kDa polypeptide	EF	F_1_ < B73 ≈ Mo17	UDO	Up
GRMZM2G017606	SHI	SHI	F_1_ < B73 ≈ Mo17	UDO	Up
GRMZM2G039889	Cold acclimation protein	–	F_1_ < B73 ≈ Mo17	UDO	Up
GRMZM2G088309	Drooping leaf	YABBY	F_1_ < B73 ≈ Mo17	UDO	Up
GRMZM2G078077	TCP domain protein	TCP	Mo 17 < F_1_ < B73	Other	Up
GRMZM2G404426	Zinc finger	Znf-PHD	B73 < F_1_ < Mo17	Other	Up

^a^ Annotations are based on Ensembl gene descriptions at maizesequence.org, gene build5b.60; ^b^ FDR ≤ 0.001 and the absolute value of log_2_Ratio ≥ 1; ^c^ Gene expression of F_1_ hybrid is classified into multiple patterns. HPD indicates high-parent dominance; LPD, low-parent dominance; ODO, over-dominance; UDO, under-dominance; ND, undistinguishable model from additivity and non-additivity; ^d^ reference Eveland *et al.* (2010) [52]. NS, Not significant.

### 2.7. A Significant Number of Genes Were Expressed in Only One Inbred Line or Absent in both Inbred Lines

A total of 5660 (24.8%) genes with no detectable expression in one inbred line or both two inbred lines were identified (Table 7). In other word, there are a significant number of genes that displayed presence–absence expression patterns. Among these genes, 46.4% (2624 of 5660) genes exhibited an expression pattern that was present in B73, and absent in Mo17, while some other 34.8% (1971 out of 5660) genes exhibited a similar expression pattern that present in Mo17, and absent in B73 (Table 7 and Table S13). Moreover, these genes expressed in only one inbred line also displayed presence–absence expression patterns in their hybrid. Specifically, regarding the genes present in B73 and absent in Mo17, the ratio of genes present to genes absent in their hybrid was approximately 0.56:1, while the ratio of genes present to genes absent in their hybrid was approximately 1.31:1 for other genes present in Mo17 and absent in B73. In combination, these results suggested that the majority of genes that were expressed in only one inbred line exhibited parental effects on gene expression levels, and presence–absence expression patterns of some genes may be related with presence/absence variations in maize genes [53] (See the following analysis). Surprisingly, it was found that 18.8% (1065 of 5660) genes were not expressed in the two inbred parents. However, these genes were expressed in their hybrid (Table 7 and Table S13). Additionally, nine genes expressed in only one inbred line were also found in previous study by Stupar *et al.* [14], and these results in two studies were consistent. For instance, GRMZM2G037255 (corresponding accession #CF629797) and GRMZM2G152258 (accession #BM073080) absent in both inbred lines in our study were not detected in both B73 and Mo17 by PCR in the study of Stupar *et al.* [14] (Table S13). However, the other 106 genes were expressed in only one inbred in Stupar’s study [14], and were expressed in both B73 and Mo17 in present study.

**Table 7:** Genes with no detectable expression in one inbred line or both two inbred lines.

Class	No. of Genes Present in B73, Absent in Mo17	% of Genes	No. of Genes Present in Mo17, Absent in B73	% of Genes	No. of Genes Absent in both B73 and Mo17	% of Genes
No. of genes present in their hybrid	942	16.6	1117	19.7	1065	18.8
No. of genes absent in their hybrid	1,682	29.7	854	15.1	0	0
Total	2,624	46.4	1971	34.8	1065	18.8

### 2.8. Analysis of Presence/Absence Variation (PAVs) Genes Action in Maize Hybrids

Presence/absence variations (PAVs) have been described in maize genes [53], and most of the PAVs reflected true differences in gene content between the B73 and Mo17 genomes in recent research. Because of the availability of a complete list of PAV genes identified by Lai *et al.* [54], the B73 × Mo17 cross was first examined regarding PAV genes between the two parental lines and its relation to different gene expression among the three genotypes. As shown in Table S14, there were 104 PAV genes between the two parental lines, and only 37 PAV genes (35.6%) were mapped by identified tags in three libraries. Interestingly, most of these mapped genes products were hypothetical proteins, and the other 55.8% (58 of 104) of genes were unknown proteins except for nine genes including terpene synthase and ZCN20 (Table S14). Of these mapped genes, 45.9% (17 of 37) genes were expressed in only B73. Six genes expressed in only one inbred line were also not expressed in their hybrid. What was more puzzling is there were three genes which were not expressed in the two inbred parents, nevertheless, expressed in their hybrid. Interestingly, among the 37 PAV genes, the expression levels (TPM) of 30% genes in F_1_ hybrid was the same as that of B73 or Mo17, and 50% showed higher or lower expression in F_1_ hybrid than both the parental lines (Table S15). However, only five PAV genes were identified with significantly differential expression with HPD or LPD (Table S14).

## 3. Discussion

In this study, we assayed genome-wide patterns of gene expression of the maize ear at an early flower differentiation stage among two maize elite inbred lines (B73 and Mo17) and their F_1_ hybrid (B73 × Mo17) using Solexa’s digital gene expression (DGE) system, a tag-based novel high-throughput transcriptome deep sequencing method. Given the nature of the DGE system, we have pooled biological replicates from three varieties for each group to make representative samples for deep sequencing analysis. We obtained a sequencing depth of approximately 4.2 million tags per library (Table 1) and found 22,789 genes expressed collectively except for putative new transcripts found in the study. Levels of some genes not expressed in the present study were responsive to abiotic stress, e.g., aquaporin PIP1-6 gene (GRMZM2G136032), MADS-box transcription factor 14 gene (GRMZM2G137510), beta-fructofuranosidase 1 precursor gene (GRMZM2G394450), *etc.*, were induced by heavy metal Pb treatment in one recent study [55]. Interestingly, we found evidence for bidirectional transcription in all datasets. By comparison of all libraries, the ratio of sense to antisense strands of the transcripts was approximately 1.3:1, which suggested that not only a high number of antisense expressions, but also the transcriptional regulation in the young ear development acted most strongly on the sense strand. A similar observation was also reported in a recent study [56]. Furthermore, approximately 20% of genes with no detectable expression in one inbred line were identified. These genes may exhibit parental effects on gene expression levels.

Based on our digital gene expression analysis, approximately 37.8% of genes exhibited differential expression between every two genotypes in the B73 × Mo17 hybrid cross. QPCR validation was performed both on the same pooled material that was used for deep sequencing and on independent RNA extractions from each sample, and almost all confirmed the direction of change detected by DGE analysis (Figure 3). These different genes exhibited additive and non-additive expression patterns (Figure 2). A small fraction of DGs (8.9%, 767 of 8621) exhibited a mode of gene action that was indistinguishable from additivity, which is similar to recent studies in maize [14,23,25,31]. Several studies reported more nonadditively expressed genes, including many with F_1_ expression levels outside the parental range [11,12,13,23,27]. Among those nonadditively expressed genes, the proportion of genes with clear over and under-dominant gene action were 12.6% and 22.8%, respectively, which is similar to results from prior studies [12,15,16,31,57]. Additionally, we further compared the differences of modes of gene action between Swanson-Wagner’s study [31] and the present study. A total of 8621 different gene BLAST searches were performed using 1367 ESTs as queries. Four hundred and twenty-one different genes were matched by 547 ESTs with high-scoring segment pairs (Table S15), but only a small number of these genes (75/421) had the same mode of gene action between the two studies. Thus, different global expression patterns in different tissues or developmental stages might prevail. Our results support that multiple molecular mechanisms (dominance and overdominance modes) contribute to heterosis, which is consistent with previous reports [21,31].

No consensus has yet been reached about the genetic basis of heterosis [58]. However, some mechanisms were supported by the observations that sequence polymorphism in promoter alleles between inbred lines preferentially occurred in those differentially transcribed genes [16]. When two alleles are exposed to a common *trans*-acting factor, *cis*-elements in hybrids might differentially interact with gene regulators, resulting in allele-specific gene expression [14,16,59]. This undoubtedly is one of the causes of gene-expression changes in hybrids. There was evidence that phenotypes in hybrids resulted from the dosage effect of such regulatory genes [60]. In this study, we found 424 putative TFs, exhibiting differential expression in the hybrid compared with either parent, in agreement with recent studies [16]. Remarkably, about 9.2% of these TFs were also differentially expressed in *ra3* mutants (Table 6) and many of the differentially expressed genes that could be mapped onto metabolic pathways were associated with primary carbohydrate biosynthesis and degradation, respiration, and energy production as well as redox and nitrogen cycling processes [52]. In conclusion, the expression of TFs in maize hybrids might be important for allele-specific gene expression in heterosis. Another important finding in a recent study is that many SNPs, indel polymorphisms (IDPs) and PAV genes identified between the B73 and Mo17 genome [54] were consistent with the occurrence of insertion/deletion (indel) variants in 5' regions between the alleles of genes that are differentially expressed in different rice strains [16]. We also analyzed the expression of all 104 PAV genes between the two parental lines by DGE data. Of these genes, 67 PAV genes (64.4%) did not express in both B73 and Mo17, possibly because these genes were nonfunctional. For another 37 PAV genes, most (33/37) of their gene products were hypothetical proteins, unknown proteins, or no significant BLAST hits were obtained by using an *e*-value cutoff of 1 × 10^−5^, and almost 50% of these genes were expressed in both inbred lines and their hybrid. Specially, eight PAV genes were expressed only in one inbred line and their hybrid, which possibly contributed to heterosis, because the phenomenon conformed to the previous assumption that inbred lines with large differences in gene content could complement one another [54]. Moreover, the expression levels of approximately 30% (9 of 37) of these genes in the F_1_ hybrid was the same as that of B73 or Mo17, and 50% showed higher or lower expression in F_1_ hybrid than both the parental lines. Interestingly, only five PAV genes were identified with significantly differential expression in the hybrid. It suggests that the expression of only a part of PAV genes was consistent with the complementation hypothesis. In conclusion, it is unlikely that heterosis is the result of any single mechanism [58,61].

## 4. Experimental Section

### 4.1. Plant Growth and RNA Isolation

The hybrid corn combination, B73 × Mo17 (F_1_ and its parental lines, B73 and Mo17), was originally obtained from Thomas Lübberstedt (Iowa State University, Ames, IA, USA). The materials were offered for high-throughput sequencing and quantitative real time PCR (qRT-PCR) analysis. The inbred lines were cultivated at the experimental station of Sichuan Agricultural University, Chengdu, for seed propagation of the inbreds, and for production of B73 × Mo17 hybrid seed. Kernels of the combination were grown in environmentally controlled growth chambers that provided 15 h of light (25 °C) and 9 h of dark (20 °C) as described previously. Light intensity was ≈650–800 μmol·m^−2^·s^−1^. Ears of 10 random healthy plants at early flower differentiation stages were manually collected as a pool for each genotype as described previously [62]. In brief, ears at stage 3 (three stamen primordia and one pistil primordium can be observed) were collected according to the plant features (number of visible leaves, leaf age index, number of unfolded and folded leaves) combined with microscopic observation. At stage 3, the number of visible leaves, leaf age index, and the number of unfolded and folded leaves are 19, 65%, 13.7 and 5, respectively. Morphological observations of ears at stage 3 in two inbred lines and their F_1_ were almost the same as that in another inbred line X178 reported previously [62]. After separately grinding meristems in liquid nitrogen, RNA were extracted for constructing three digital gene expression libraries, and quantitative real-time PCR validation from ≈5 g of frozen tissue by using TRIzol reagent (Invitrogen, Carlsbad, CA, USA) according to the manufacturer’s instructions.

### 4.2. Digital Gene Expression Library Preparation and Sequencing

Tag library preparation for the three genotypes (B73, Mo17 and their F_1_) was performed in parallel by using the Illumina gene expression sample preparation kit as described previously [63]. An extract of 6 μg of total RNA was obtained and treated with Oligo (dT) magnetic bead adsorption to purify mRNA. Oligo (dT) was then used as a primer to synthesize the first- and second-strand cDNA. The 5' ends of tags were generated by two endonucleases *Nla*III or *Dpn*II. The bead-bound cDNA was subsequently digested with restriction enzyme *Nla*III, which recognizes and cuts off the CATG sites. The fragments apart from the 3' cDNA fragments connected to Oligo (dT) beads were washed away and the Illumina adaptor 1 was ligated to the sticky 5' end of the digested bead-bound cDNA fragments. The junction of Illumina adaptor 1 and CATG site is the recognition site of *Mme*I, which is a type of Endonuclease with separate recognition and digestion sites. It cuts 17 bp downstream of the CATG site, producing tags with adaptor 1. After removing 3' fragments with magnetic beads by precipitation, Illumina adaptor 2 was ligated to the 3' ends of tags, acquiring tags with different adaptors at both ends to form a tag library. After 15 cycles of linear PCR amplification, 95 bp fragments were purified by 6% TBE PAGE Gel electrophoresis. After denaturation, the single-chain molecules were fixed onto the Illumina Sequencing flowcell. Each molecule grows into a single-molecule cluster sequencing template through *in situ* amplification. The four types of nucleotides were labeled by four colors, and added to perform sequencing by synthesis (SBS) [64]. Each tunnel will generate millions of raw reads with sequencing length of 35 bp.

### 4.3. Quantitative RT-PCR and Gene Expression Analysis

In order to verify a sample of genes that exhibited statistically significant differential expression in the analysis of DGE data, we used quantitative real time PCR analysis. The RNA samples used for the qRT-PCR assays were the same as for the DGE experiments from 10 biological replicates. First, 1 μg of RNA was treated with RNase-free DNase (Promega, Madison, WI, USA), and cDNA was synthesized with PrimeScript RT reagent kit (TaKaRa, Tokyo, Japan). Then, qRT-PCR of 30 differentially expressed genes (Table S16), which were involved in arginine and proline metabolism, pyruvate metabolism or other genes, were performed using the SYBR *PremixExTaq*™ protocol (TaKaRa, Tokyo, Japan) on an Applied Biosystems 7500 Real-Time PCR System (Applied Biosystems, Foster City, CA, USA). For each sample, measurements were performed in triplicate, and the average cycle thresholds (*C*_t_) were used to determine fold-change. 18S rRNA was employed as an endogenous control. The results were calculated using the 2**^−∆∆^***^C^*^t^ method [65].

### 4.4. Analysis and Mapping of Digital Gene Expression Tags

Raw sequencing image data were transformed by base calling into sequences. These raw data reads were stored in FASTQ format, and their analysis conducted as described by Qin *et al.* [63]. In brief, prior to mapping to the reference database, all sequences were filtered to trim the 3' adaptor sequence, filter empty tags (reads with only 3' adaptor sequences but no tags) and low-quality tags containing Ns, and remove tags which are too long or too short. A virtual library containing all possible CATG + 17 base-length sequences of the maize genome database (AGPv2, release 5b.60) [43] was utilized. All clean tags were mapped to the reference sequences and a mismatch of only 1 bp was considered. Clean tags that were mapped to the maize genome reference sequences from multiple genes were filtered. The remaining clean tags were designed as unambiguous clean tags. The expression level of each gene was estimated by the frequency of clean tags and then normalized to TPM (number of transcripts per million clean tags) [66], which is a standard method and extensively used in DGE analysis [67]. KOG functional classification, Gene Ontology (GO), pathway annotation and enrichment analyses were based on the NCBI COG (http://www.ncbi.nlm.nih.gov/COG) [68], Gene Ontology Database (http://www.geneontology.org/) [69] and KEGG pathway (http://www.genome.jp/kegg/) [70], respectively. When we investigate pathways in which DGs were involved and enriched, *q*-value was used for aided identification according to the previous description [47].

### 4.5. Identification of Differentially Expressed Genes

To examine differential expression across samples (B73, Mo17 and their hybrid), the number of raw clean tags in each library was normalized to TPM to obtain normalized gene expression levels. Detection of different tags across samples were performed as previously described [71]. The false discovery rate (5%) is controlled by the Benjamini and Hochberg’s procedure [72]. After multiple testing between pairwise comparisons, we use “FDR ≤ 0.001 and the absolute value of log_2_Ratio ≥ 1” as the threshold to judge the significance of gene expression difference. More stringent criteria with smaller FDR and bigger fold-change values were used to identify different genes.

In the present study, the same strategy was performed in a linear-in-genotype contrast when F_1_ genotype was compared to the two parental lines as described by Zhang *et al.* [16]. The genes with “FDR ≤ 0.001 and the absolute value of log_2_Ratio ≥ 1” were regarded as non-additivity, when the genes with “FDR > 0.001 and the absolute value of log_2_Ratio < 1” were regarded as not statistically significantly different from additivity. To classify the genes further, the high-parent dominant genes and low-parent dominant genes were identified from the non-additive group based on the criterion, that the F_1_ genotype was significantly different from one parent and not significantly different from another parent. From the non-additive group, the expression of genes was identified as over- or under-dominant, when expression in the F_1_ genotype was significantly higher or lower than in both inbred parents, respectively.

## 5. Conclusions

Our analysis revealed 17,128 genes expressed in these three genotypes and 22,789 genes expressed collectively in the present study. Approximately 38% of the genes were differentially expressed in early maize ear inflorescences from heterotic cross, including many transcription factor genes and some presence/absence variations (PAVs) genes, and exhibited multiple modes of gene action. Additionally, a significant number of genes were expressed in only one inbred line or absent in both inbred lines. Comparison of the differences of modes of gene action between previous studies and the present study revealed only small number of different genes had the same modes of gene action in both maize seedlings and ear inflorescences, it might of be an indication that, in different tissues or developmental stages, different global expression patterns might prevail, which might nevertheless be related to heterosis. Our results support the hypothesis that multiple molecular mechanisms (dominance and overdominance modes) contribute to heterosis.

## Supplementary Files

Supplementary File 1
