# Supplementary material for: Heterosis in Early Maize Ear Inflorescence Development: A Genome-Wide Transcription Analysis for Two Maize Inbred Lines and Their Hybrid
Source: Int J Mol Sci. 2014 Aug 11;15(8):13892–915. doi: 10.3390/ijms150813892 (PMC4159830; doi:10.3390/ijms150813892)

Supplementary Information

Figure S1. Distribution of total and distinct clean tags over different tag-abundance categories. (**a**) Distribution of total clean tags. Normalized tag copy number was calculated by dividing tag counts for each gene with the total number of tags generated for each library and are presented per one million transcripts. Numbers within square brackets indicate the range of copy numbers for a specific category of tags. Numbers within parentheses represent the total tag copy number for all clean tags in that specific category; (**b**) Distribution of distinct clean tags. Numbers within square brackets indicate the range of copy numbers for a specific category of tags. Numbers within parentheses represent the total types of tags in that specific category.

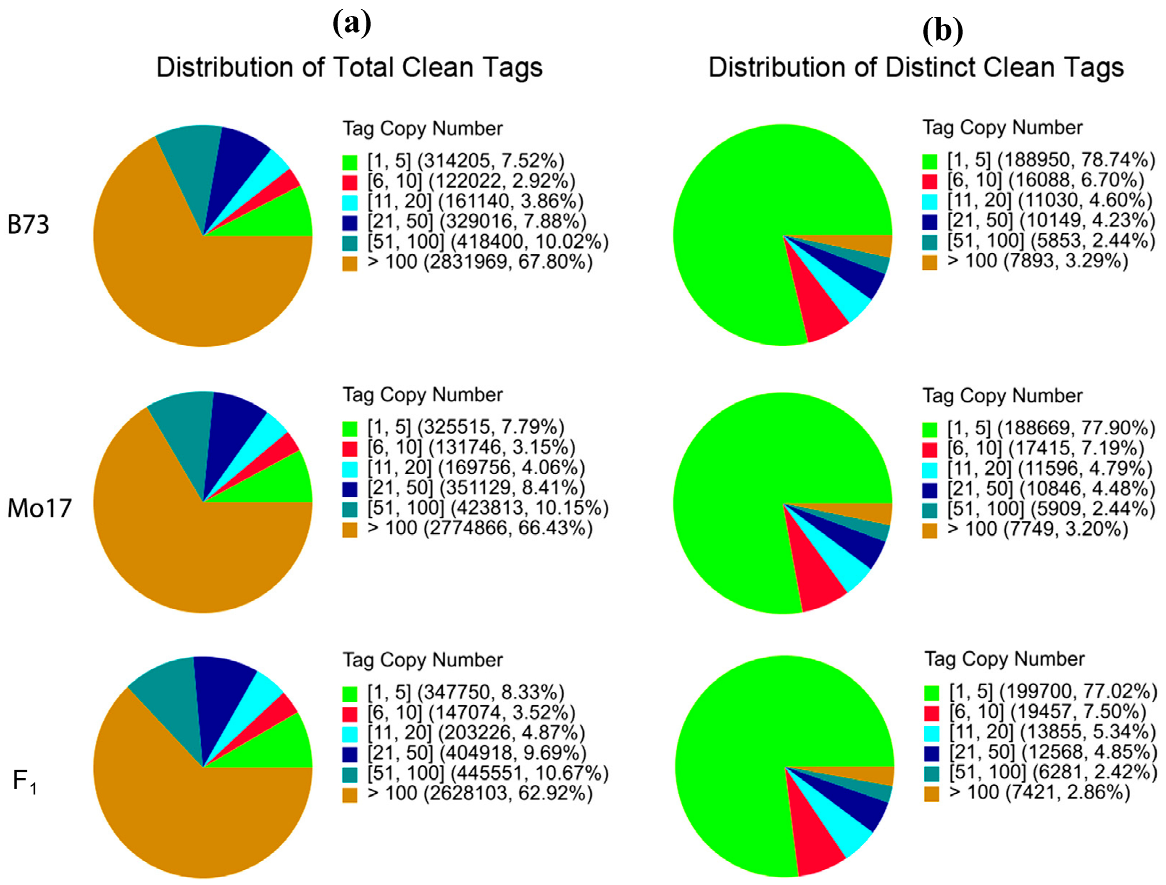

Figure S2. Saturation of digital gene expression (DGE) libraries. Diagrams on the left (**a**) presents the relationship between the percentage of genes identified and total tag number in the library of B73, Mo17 and F_1 (B73 × Mo17); Saturation analysis of capacity of libraries in right diagrams (**b**) showed that new emerging distinct tags were gradually reduced with increasing of total sequence tags when the number of sequencing tags was big enough.

(**a**) (**b**)

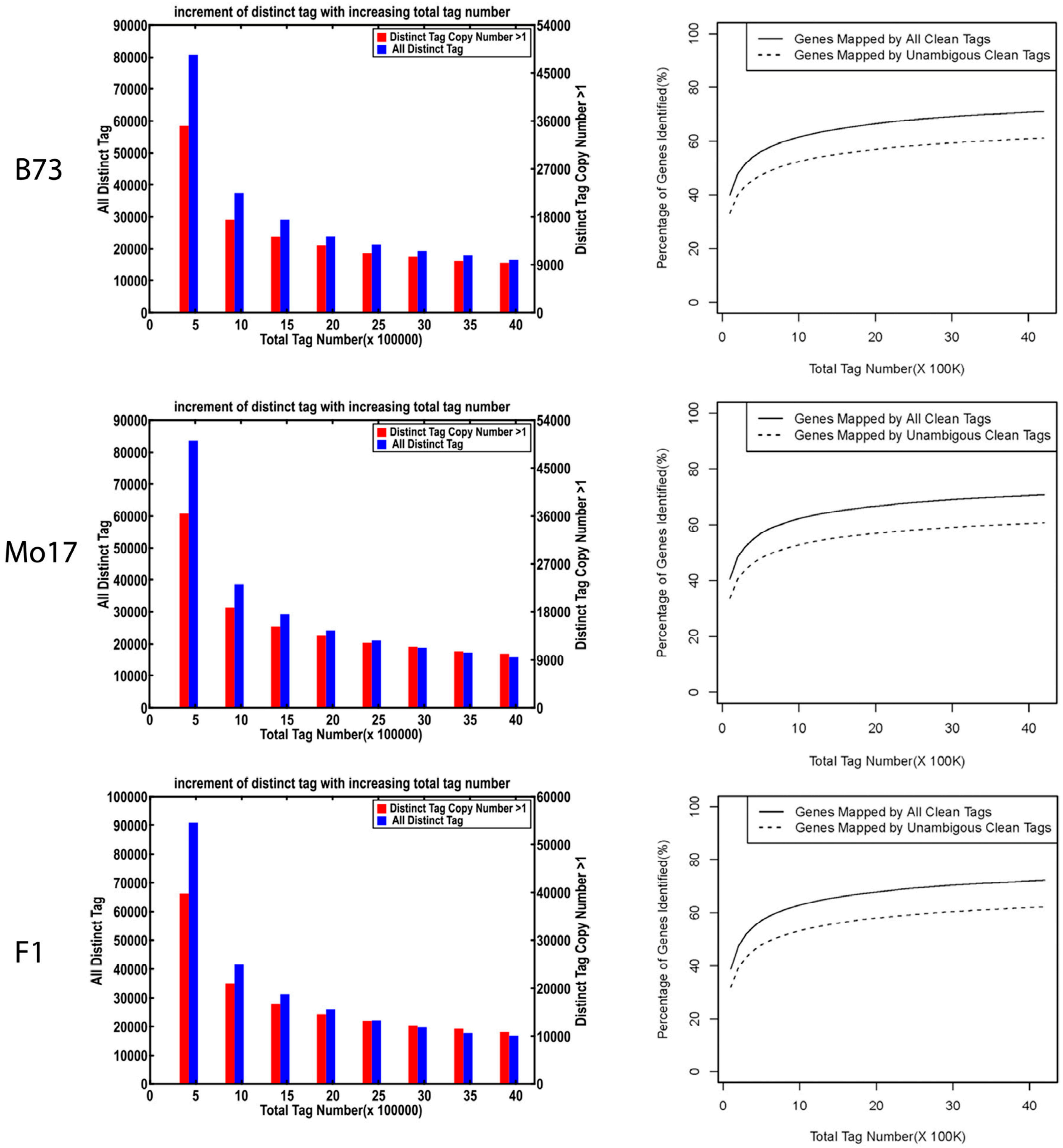

Figure S3. The level of gene expression for each gene between inbred parents or between parental lines and their F_1 hybrids. Gene expression level was determined by calculating the number of unambiguous tags for each gene and then normalizing to TPM (transcript copies per million tags). (**a**) B73, (**b**) Mo17 and (**c**) F_1 (B73 × Mo17).

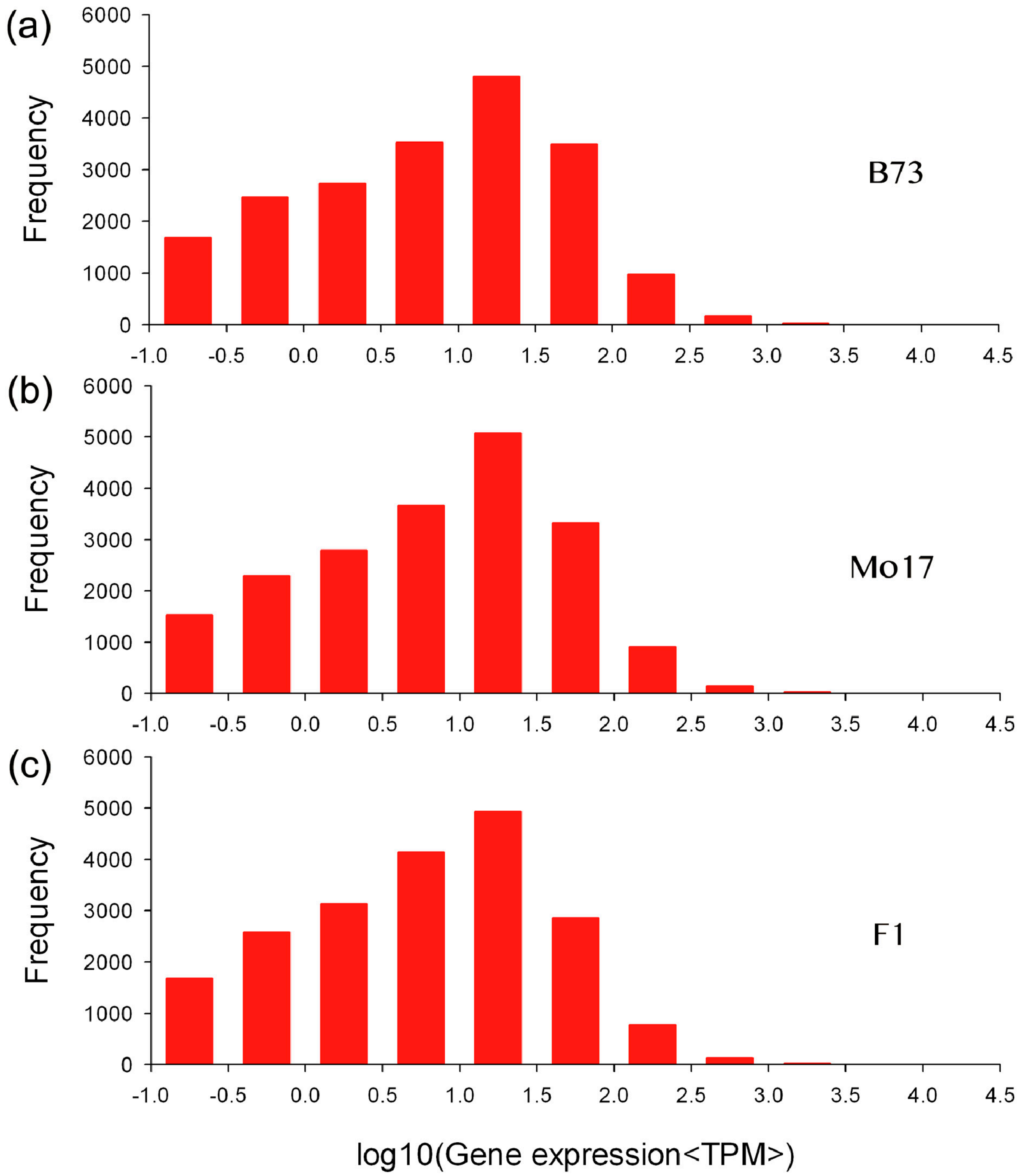

Figure S4. Differential expression analyses of tags and genes by DGE. The expression level for each tag and genes was included in the volcano plot (Figures a and b, respectively). "Not DETs" and "Not DEGs" indicate "not detected expression tags" and "not detected expression genes", respectively. For Figures (**A**–**F**), the x-axis contains Log_{10} of transcript per million of one ample and the y-axis indicates Log_{10} of transcript per million of another sample. Limitations are based on PDF < 0.001, and the absolute value of Log_2(B/A) is greater than 1. F, P, and M stand for F1 (B73 × Mo17), B73, and Mo17, respectively.

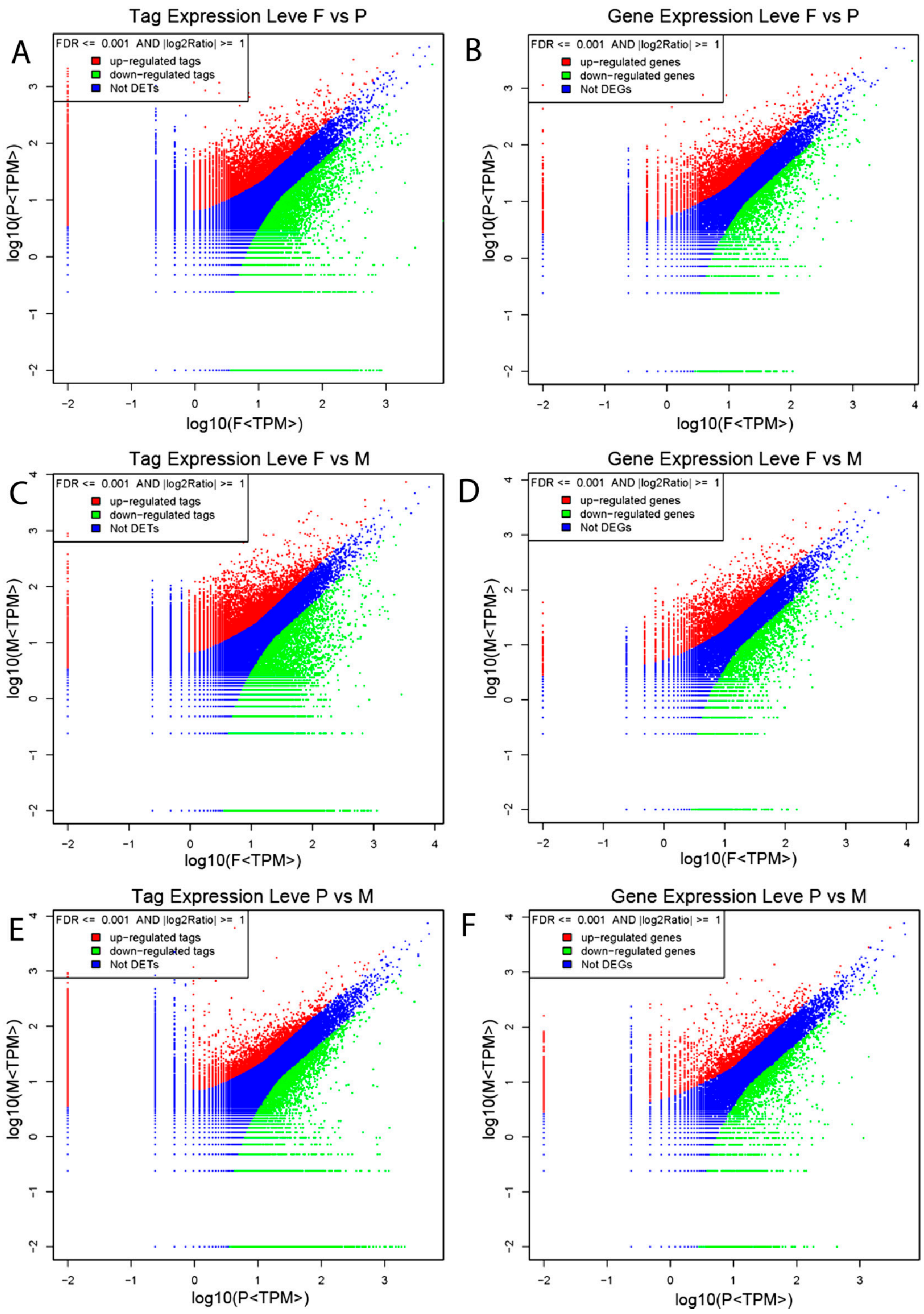

Supplement: Supplementary File 1 [file ijms-15-13892-s001.zip › ijms-56986-SI-final.pdf]
